# Age and magnitude of acetabular correction impair bone healing after triple pelvic osteotomy

**DOI:** 10.1007/s00402-023-04966-z

**Published:** 2023-07-08

**Authors:** Daniel Dornacher, Bernd Lutz, Mirco Sgroi, Thomas Caffard, Heiko Reichel

**Affiliations:** Department of Orthopedics, University of Ulm, Oberer Eselsberg 45, 89081 Ulm, Germany

**Keywords:** Hip preservation surgery, Triple pelvic osteotomy, Bone healing, Micromotion, Non-union

## Abstract

**Introduction:**

The aim of this examination was to assess, which risk factors impair bone healing after triple pelvic osteotomy (TPO) in the treatment of symptomatic hip dysplasia.

**Methods:**

A consecutive series of 241 TPO was reviewed retrospectively. Of these, a set of five postoperative radiographs was available, performed in a standardized regimen in the first year after surgery. Two experienced observers had to agree on the existence of a non-union on the radiographs obtained 1 year after TPO. Both observers measured the lateral center edge angle (LCEA) and acetabular index (AI) on all radiographs. Besides patient-specific risk factors, the magnitudes of acetabular correction and the amounts of a detectable slight change in acetabular correction were assessed. Binary logistic regression analysis and chi-squared test were used to detect the impact of the risk factor on bone healing.

**Results:**

A total of 222 cases were left for further examination. In 19 of these, at least one osteotomy was not healed completely one year after surgery. Binary logistic regression showed a significant relationship between the risk factors “age” (*p* < 0.001; odds ratio (OR) 1.109 (95% CI 1.05–1.18)) as well as “magnitude of acetabular correction (LCEA)” (*p* = 0.01; OR 1.087 (95% CI 1.02–1.16)) and non-union. Pearson’s chi-square test showed a relationship between the risk factor “wound healing disorder” and non-union (*p* < 0.001). LCEA and AI showed a slight increase from the first to the last follow-up (observer 1: 1.6° and 1.3°, resp.), but regression analysis for the risk factor “amount of postoperative change of acetabular correction (LCEA, AI)” did not show statistically significant values.

**Conclusion:**

The age at surgery and the magnitude of acetabular correction negatively influenced the healing progress of the osteotomy sites. The amount of a slight postoperative change of LCEA and AI did not correlate with a non-union.

## Introduction

In the treatment of hip dysplasia in the adolescent and the adult, triple pelvic osteotomy (TPO) as described bx Tönnis and Kalchschmidt, is a well-established procedure [1, 2]. The acetabular fragment is mobilized by osteotomies of the ischium, pubis, and ilium, allowing a precise three-dimensional reorientation of the acetabulum. The mobility of the acetabular fragment provides a powerful tool for the orthopedic surgeon. But the iatrogenically created discontinuity of the hemipelvis has to be considered temporally unstable, as long as sufficient healing of the osteotomies has occurred [3]. There is evidence, that the incidence of non-unions after pelvic osteotomies influence patient satisfaction [3–5]. On the other hand, there is a lack of studies aiming at the risk factors leading to a disturbance of bone healing after TPO. Furthermore, in our regular analyses of the radiographic follow-up, it became apparent that the acetabular fragment was subject to a very slight settlement behavior over the course of a year. The emergence of this phenomenon might be supported by a rather close-meshed follow-up regimen of our patients after surgery. Since the right level of stability is key to successful bone healing, the above-mentioned settlement behavior of the acetabular fragment might play a role in this matter. To the best of our knowledge, this has not been subject to scientific work so far.

We hypothesized, that several patient-related risk factors, as well as the magnitude of acetabular correction and the amount of a postoperative change of acetabular correction, were related to a disturbance of bone healing.

## Materials and methods

**Fig. 1:**
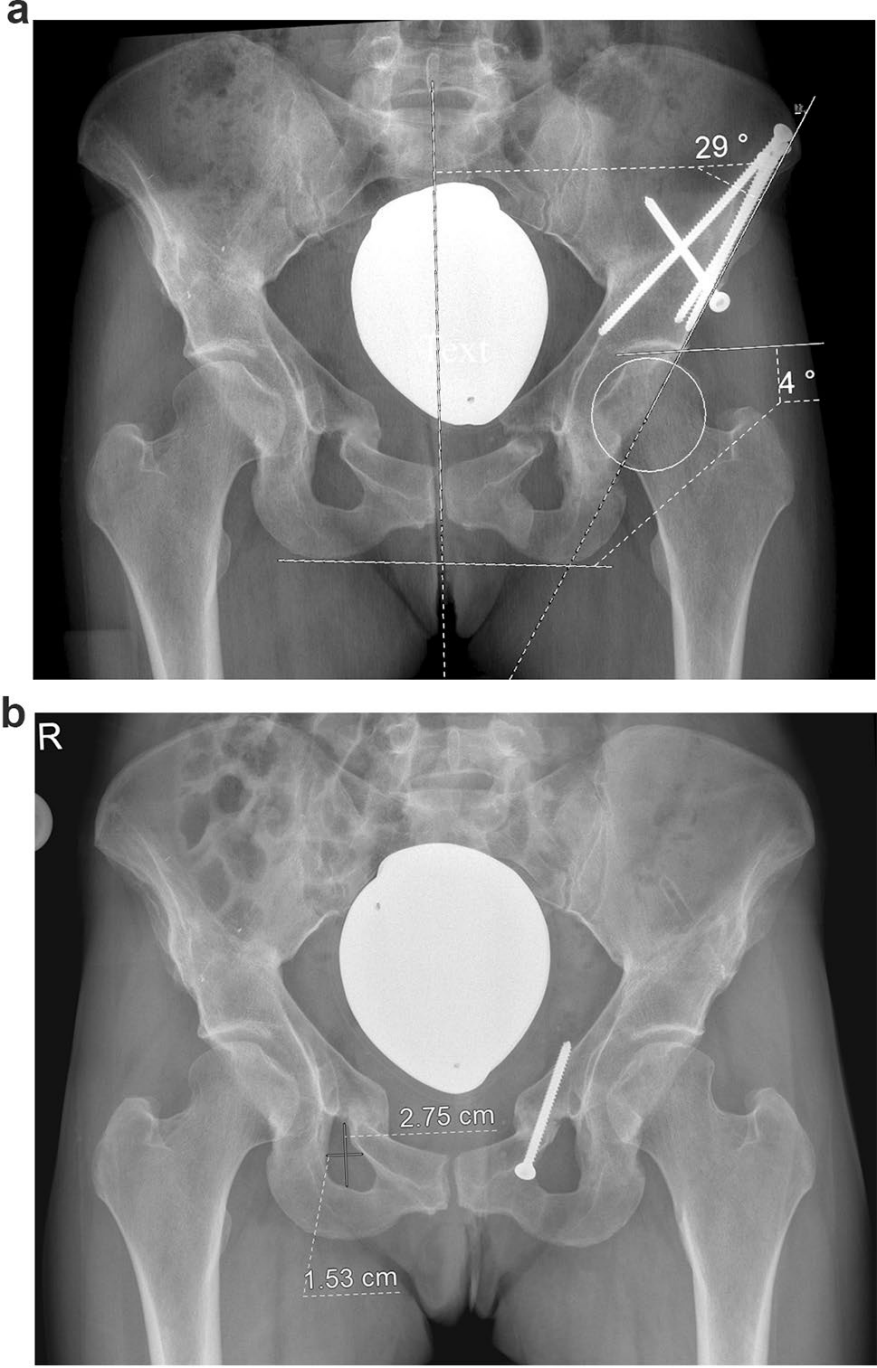
**a** Pelvic radiograph of a female, performed one year after TPO. Surgery around the left hip took place at the age of 38 years, the right hip had been treated two years earlier. BMI was 20.5, the patient consumed 20 cigarettes a day. TPO was indicated due to symptomatic hip dysplasia with an LCEA of 14° and an AI of 18°. A rather large acetabular correction had to be performed, resulting in an LCEA of 29° and an AI of 4° (Δ LCEA pre- to postoperative: 15°, Δ AI pre- to postoperative: 14°). The patient complained about groin pain, different from the joint-related pain she experienced before TPO. A test infiltration of the left hip joint did not relieve the pain, thus the non-union of the pubic osteotomy was considered the cause of the complaints. The measurement routine of LCEA and AI is displayed at the left hip. Please see the explanation in detail in the main text under the heading “Measurement routine and definition of a non-union”. **b** Revision surgery of the pubic osteotomy was performed 1 year after TPO, simultaneously with the removal of the fixation screws from the ileum, including autogenous cancellous bone grafting from the iliac crest and osteosynthesis of the pubic bone with a fully threaded lag screw. The assessment of the height-width relation is demonstrated on the right obturator foramen. Please see the explanation of the measurement routine in the main text under the heading “Radiographic management and follow-up regimen”

For this examination, a consecutive series of 241 TPO was reviewed retrospectively. All procedures were performed from January 2015 to December 2019 in our orthopedic department in a total of 206 patients (178 female, 28 male) (for demographics and anthropometric data in detail please see Table 1). The patients were predominantly referred to our outpatient department due to symptomatic hip dysplasia. When the patients reported a load-dependent hip-pain which did not respond to a series of specific physiotherapy including muscle activation and strengthening, surgical treatment was recommended. Decision-making was supported by deformity analysis performed on a plain pelvic radiograph (see below). A set of radiographic parameters [Lateral center edge angle (LCEA), acetabular index (AI), anterior and posterior wall index (AWI and PWI)] allowed a comprehensive assessment of acetabular orientation. The surgical sequence was performed in a highly-standardized manner by two surgeons, according to the method described by Tönnis and Kalchschmidt [1, 2]. During the operation, acetabular reorientation was guided by fluoroscopy, which has been proven to be reproducible and reliable [6–8]. The acetabular fragment was fixed with 4.5 mm fully threaded steel screws, predominantly in a specific pattern using four screws: the objective was to spread three screws widely over the osteotomy and to place one screw perpendicularly (Fig. 1). In a recently published finite element analysis, it has been shown that the predominantly used fixation pattern with widely spread screws over the osteotomy and a perpendicular fixation generated improved stability [9]. To minimize the operative trauma of TPO and of the implant removal, screw fixation of the pubic osteotomy was not performed.

**Table 1:** Anthropometric data and patient-related risk factors

	Total	consolidation	non-union	Asymptomatic non-union	Revision due to non-union
Number of cases (*n*; % of total)	222	203 (91.4%)	19 (8.5%)	11 (5.0%)	8 (3.6%)
Female/male (*n*)	194/28	175/28	19/0	0/11	0/8
Age [years] mean (min–max; SD)	26.3 (9.7–48.0; 8.49)	25.6 (9.7–45.9; 8.17)	33.0 (15.8–48; 8.74)	32.1 (15.8–48; 9.66)	34.3 (22.6–42.8; 7.03)
Body height [cm] mean (min–max; SD)	167.8 (138–203; 8.58)	167.9 (138–203; 8.88)	166.7 (158–175; 4.12)	166.7 (160–175; 3.96)	166.7 (158–174; 4.31)
Body weight [kg] mean (min–max; SD)	69.0 (33–122; 16.12)	69.0 (33–120; 16.03)	69.5 (40–122; 17.02)	70.85 (40–122; 20.19)	67.6 (53–84; 11.2)
BMI [kg/m^2^] mean (min–max; SD)	24.4 (14.5–45.4; 4.87)	24.4 (14.5–38.7; 4.73)	25.1 (15.6–45.4; 6.17)	25.7 (15.6–45.4; 7.27)	24.4 (18.8–30.9; 4.20)
Nicotine consumption (*n*; %)	61 (27.7%)	55 (27.1%)	6 (31.6%)	2 (18.2%)	4 (50%)
Trauma (stumbling or falling in the first 12 weeks)	10 (4.5%)	8 (3.9%)	2 (10.5%)	2 (10.5%)	0
Wound healing disorder	7 (3.2%)	3 (1.5%)	4 (21.1%)	3 (27.3%)	1 (12.5%)
ASA-classification (*n*)	ASA 1: 118; ASA 2: 96 ASA 3: 8	ASA 1: 112 ASA 2: 84 ASA 3: 7	ASA 1: 6 ASA 2: 12 ASA 3: 1	ASA 1: 6 ASA 2: 4: ASA 3: 1	ASA 2: 8

### Postoperative mobilization

After surgery, the patients followed a standardized mobilization protocol, supervised by experienced physiotherapists. In the first few days after surgery, all patients were verticalized successfully, using underarm sticks with partial weight bearing on the operated limb [20 kilograms (kg)]. All patients received the first radiographic control immediately after surgery. Five days later, the second pelvic radiograph was performed. When the comparison with the immediate postoperative image showed no relevant discrepancies, one week after surgery, almost all patients were referred directly to our in-house rehabilitation center to receive 3 weeks of specific rehabilitation measures. This was followed by outpatient physiotherapy on prescription. When the third radiographic follow-up 6 weeks after surgery did not reveal any relevant changes in acetabular correction, partial weight bearing was increased step-by-step (10 kg per week) until full weight bearing was reached.

### Radiographic management and follow-up regimen

Every patient received a standardized antero-posterior (AP) pelvic radiograph in the radiological department of our institution with the purpose of deformity analysis and planning of the correction. The radiograph was produced in the supine position, with a film-focus distance of 1.15 m, the beam centered between the symphysis and a line connecting the anterior superior iliac spines, both legs fully extended and 15° inwardly rotated. After fluoroscopically-guided acetabular reorientation and osteosynthesis, at the very end of the surgery, an AP pelvic radiograph was performed on the operation table, technically executed as described above. The complete radiographic follow-up comprised pelvic radiographs 5 days, 6 weeks, 12 weeks and 1 year after the procedure. All postoperative radiographs were performed in an identical manner in the radiological department of our hospital. The radiographs were archived in the picture archiving and communication system of our institution (PACS, GE Centricity Universal Viewer Version 6.0, General Electric Healthcare, Chalfont St Giles, UK). For each treated case, six pelvic radiographs were available for the assessment of acetabular orientation (preoperative, day of surgery, 5 days after surgery, 6 weeks, 12 weeks, and 1 year after surgery). Consequently, in the postoperative follow-up, a possible change in acetabular correction was attributable to a specific postoperative period. Quality control of the radiographs comprised a check for relevant malrotation or tilt. In the female cases, the assessment of constant distances from the symphysis to the tip of the coccyx or the sacrococcygeal joint was not possible due to the gonad shielding. In these cases, we opted for a surrogate parameter: The width-height relation of the obturator foramen was used, acquired on the contralateral side (Fig. 1b). A percentage variation of less than 10% between the radiographs was accepted to ensure comparable conditions for the measurements. In this context, two aspects should be emphasized: First, the radiographic parameters which were used in this examination (LCEA, AI, see below) have been proven to be particularly robust in the presence of a varying pelvic orientation [8]. Second, in the very rare case of a malrotated image, e. g. due to postoperative discomfort five days after surgery, the radiograph was not accepted and redone immediately.

The following exclusion criteria were defined: (1) severe deformation of the femoral head (e.g. after Legg–Calve–Perthes disease), (2) acetabular dysplasia as a part of a syndromic disease, (3) cases with an incomplete radiographic history (one or more postoperative images missing), (4) cases with a percentage variation of more than 10% for the width–height relation of the obturator foramen, (5) cases with a relevant change of acetabular correction which underwent revision surgery.

### Measurement routine and definition of a non-union

On each of the radiographs, the lateral center edge angle (LCEA) and the acetabular index (AI) were measured by two observers (DD, BL). In contrast to parameters assessing the anteroposterior acetabular version, such as the anterior- and posterior wall index, LCEA and AI have proven their robustness against a slight tilt or rotation of the pelvic radiograph [10]. After verification of the usability and the relevant landmarks, first of all, the center of the femoral head was estimated from a circle fit to its contour. Then, the longitudinal axis of the pelvis was defined by drawing a vertical line from the processus spinosus of L5 through the middle of the symphysis. The LCEA was measured between the line from the center of the femoral head to the lateral aspect of the sourcil, and the longitudinal axis of the pelvis [11, 12]. AI was measured between a line connecting the inferior ischial tuberosities and a tangent to the most medial and most lateral aspect of the sourcil [13] (Fig. 1a).

In asymptomatic patients, non-union was defined, when the radiograph showed atrophic or hypertrophic callus formation, but no complete bone healing, twelve months after surgery. Mostly, this was observed at the pubic osteotomy. In symptomatic patients, a CT-scan around 6 to 8 months after surgery revealed atrophic or hypertrophic callus formation often at two of the three osteotomies.

### Risk factors

The patient records were reviewed with regard to the following risk factors: age, gender, body weight, body height, body mass index (BMI), nicotine abuse, an incident of stumbling or falling in the first three months after surgery (“trauma”), wound healing disorder, classification according to the American Society of Anesthesiologists (ASA). Additionally, based on the measurements of LCEA and AI, the following risk factors were observed: severeness of acetabular dysplasia (expressed by preoperative LCEA or AI), “magnitude of acetabular correction” (expressed the difference of the pre- to the postoperative LCEA or AI), “amount of postoperative change of acetabular correction” (expressed by a difference of LCEA or AI within the first twelve weeks after TPO).

### Statistical analysis

An a priori power analysis was carried out for all statistical tests to calculate the needed sample size. The intra- and interobserver reliability was quantified with intraclass correlation coefficient (ICC) using a two-way model with agreement type. The values of ICC were interpreted according to the scale described by Cicchetti: less than 0.40: poor, between 0.40 and 0.60: fair, between 0.60 and 0.75: good and greater than 0.75: excellent [14]. The results of the measurements of LCEA and AI were averaged between the first reads of the two observers. For the metrical independent variables, binary logistic regression analysis was used with the target variable “bony consolidation” (union = 0, non-union = 1). When the omnibus test of the model coefficients showed *p* < 0.05, the odds ratio (OR (Exp(B)) and the 95% confidence intervals (95% CI) were calculated. For the categorical variables, Pearson’s chi-squared test was used. A significance level of *α* = 0.05 was used for all tests. The statistical analyses and presentations were performed using SPSS Statistics, Version 29.0.0.0 (IBM, Armonk, New York, United States of America).

## Results

**Fig. 2:**
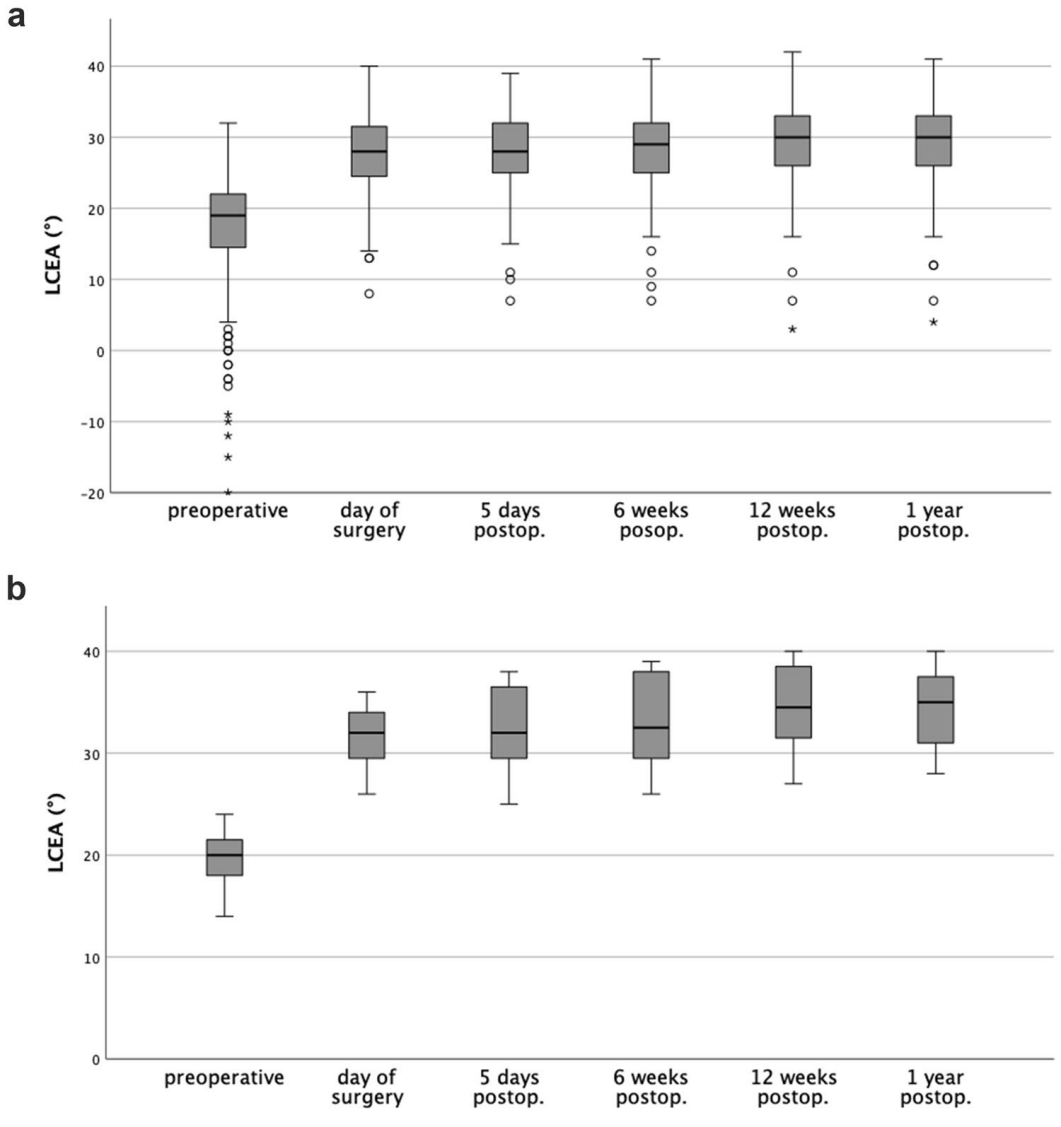
**a** The boxplots represent the distribution of LCEA for the consolidated cases (observer 1; *n* = 203 cases) (from top to bottom: maximum, 1st quartile, median, 3rd quartile and minimum). The boxplot on the very left shows the preoperative values. Postoperatively, the mean values of LCEA showed an increase of 1.5° over 1 year, suggesting a micromotion of the acetabular fragment. **b** Distribution of LCEA for the cases with non-union undergoing revision surgery (observer 1; *n* = 8 cases). The mean values of LCEA increased almost twice as high as in the consolidated cases (mean 2.75°). The largest change was measured between the sixth and the twelfth week, suggesting a slightly pronounced micromotion. This might be a sensitive period for some patients and potentially impair bone healing. When the follow-up at 6 weeks showed no change of acetabular correction, partial weight bearing was increased step-by-step (10 kg per week) until full weight bearing was reached. Nevertheless, a statistically significant relationship between the “amount of postoperative acetabular correction” and non-union could be seen neither for LCEA nor for AI

From 241 hips, 222 hips were left for further examination (194 female, 28 male hips, age at the time of the surgery 26.3 ± 8.49 years [mean, standard deviation (SD)] (for the demographics and anthropometric data please see Table 1). After application of the exclusion criteria, 19 hips were excluded due to: relevant deformation of the femoral head: 2; syndromic disease: 6; incomplete radiographic history: 3; percentage variation of more than 10% for the width-height relation of the obturator foramen: 5; relevant loss of acetabular correction leading to revision surgery: 3. On the radiograph one year after TPO, in 19 cases, a non-union of at least one osteotomy was visible. Of these, in 11 cases, the non-union was asymptomatic. In 8 cases, revision surgery was performed due to relevant symptoms which were attributable to the non-union. Correlation analysis between the two observers resulted in excellent interobserver reliability for LCEA and AI in almost all measurements (0.77–0.91), and good interobserver reliability for AI in the measurements twelve weeks after surgery (0.74). In the risk factor analysis with binary logistic regression, the omnibus test of the model coefficients showed a significance of the variables “age” (*p* < 0.001) and “magnitude of acetabular correction (LCEA)” (*p* = 0.01). The OR for the variable “age” was calculated 1.109 (95% CI 1.05–1.18), for the variable “magnitude of acetabular correction (LCEA)” OR was 1.087 (95% CI 1.02–1.16), respectively. This indicates that an increase in age by one year or an increase of acetabular correction by one degree (LCEA), increases the probability of a non-union by 11% or 9%, respectively. The further independent metrical variables [body weight, body height, BMI, “magnitude of acetabular correction (AI)”, “amount of postoperative change of acetabular correction (LCEA)” and “amount of postoperative change of acetabular correction (AI)”)] did not show a significance (*p* = 0.285–0.870) (Table 2). Pearson’s chi-square test showed a relationship between the risk factor “wound healing disorder” and non-union (*p* < 0.001), not for the risk factors “ASA”, “trauma” and “nicotine abuse” (*p* = 0.143, *p* = 0.186 and *p* = 0.675, resp.). The values for LCEA and AI from the first to the last follow-up showed a slight increase (observer 1: 1.6° and 1.3°, resp.), indicating a settlement behavior for the acetabular fragment. This slight change of acetabular correction, particularly for LCEA, was pronounced in the cases which received a surgical revision due to symptomatic non-union (Tables 3, 4) (Fig. 2). Nonetheless, binary logistic regression analysis for the risk factor “amount of postoperative change of acetabular correction (LCEA, AI)” did not show statistically significant values.

**Table 2:** The results of binary logistic regression analysis showed a significant relationship between the risk factors “age” as well as “magnitude of acetabular correction (LCEA)” and a non-union

Risk factor	Significance (*p*)	Odds ratio (OR)	95% confidence interval (CI)
Age	< 0.001	1.109	1.05–1.18
Body weight	0.870	0.99	0.97–1.02
Body height	0.575	0.98	0.93–1.04
BMI	0.845	1.009	0.92–1.11
Magnitude of acetabular correction (LCEA)	0.008	1.087	1.02–1.16
Magnitude of acetabular correction (AI)	0.773	1.024	0.87–1.12
Amount of postoperative change of acetabular correction in the first year (LCEA)	0.280	1.101	0.93–1.31
Amount of postoperative change of acetabular correction in the first year (AI)	0.314	1.102	0.96–1.25

The values of 95% CI were rounded down to two digits behind the decimal point

**Table 3:** Values of the measurements of LCEA and AI, observer 1 (DD)

Angle measurements, mean; SD [°]	Total	Consolidation	Non-union	Asymptomatic non-union	Revision due to non-union
LCEA preoperative	16.9; 9.12	17.2; 9.12	14.4; 8.60	10.5; 9.3	19.6; 2.91
AI preoperative	15.3; 8.64	14.8 8.34	20.9; 9.72	25.7; 9.5	14.3; 4.84
LCEA postoperative	27.9; 5.14	27.8; 5.22	29.4; 3.87	27.7; 3.54	31.6; 3.08
AI postoperative	3.7; 5.31	3.6; 5.32	4.0; 5.18	6.4 4.05	0.8; 4.79
Magnitude of acetabular correction (Δ LCEA pre- to postoperative)	11.0; 6.66	10.7; 6.52	15.0; 6.90	17.2; 8.21	12.0; 2.18
Magnitude of acetabular correction (Δ AI pre- to postoperative)	11.6; 6.08	11.1; 5.77	16.9; 6.77	19.4; 7.76	13.5; 2.5
Amount of postoperative change of acetabular correction in the first year (LCEA)	1.6; 2.72	1.5; 2.74	2.2; 2.40	1.8 2.66	2.75; 1.86
Amount of postoperative change of acetabular correction in the first year (AI)	1.3; 3.0	1.3; 2.97	1.1; 3.24	0.1; 2.35	2.5; 3.74

The magnitude of acetabular correction (Δ LCEA pre- to postoperative) was remarkably higher in the cases with a non-union than in the consolidated osteotomies. This was proven to be statistically significant in the regression analysis. Additionally, it is recognizable that the “amount of postoperative change of acetabular correction in the first year (LCEA)” and the “amount of postoperative change of acetabular correction in the first year (AI)” show higher values in the non-union cases compared to the consolidated osteotomies. This suggests, that a pronounced micromotion might have had an impact on the bone healing. In the statistical analysis, this could not have been substantiated with significant values. Future research will be necessary to assess to what extent a micromotion of the acetabular fragment is beneficial for osteotomy healing and when it is not anymore

**Table 4:** Values of the measurements of LCEA and AI, observer 2 (BL)

Angle measurements, mean; SD [°]	Total	consolidation	non-union	Asymptomatic non-union	Revision due to non-union
LCEA preoperative	16.2; 9.61	16.4; 9.79	14.3; 7.99	11.2; 7.95	18.5; 5.81
AI preoperative	12.2; 8.06	12.0; 7.95	14.1; 9.01	15.6; 11.1	12.1: 1.26
LCEA postoperative	25.7; 6.50	25.6; 6,57	26.1; 5.59	23.0; 4.61	29; 5.22
AI postoperative	1.9; 6.55	1.9; 6.59	1.9; 6.06	3.5; 5.82	-0.25; 5.72
Magnitude of acetabular correction (Δ LCEA pre- to postoperative)	9.5; 6.4	9.3; 6.36	11.8; 6.27	12.8; 7.40	10.5; 3.87
Magnitude of acetabular correction (Δ AI pre- to postoperative)	10.3; 5.86	10.1; 5.62	12.2; 7.74	12.1; 9.63	12.4; 3.81
Amount of postoperative change of acetabular correction in the first year (LCEA)	3.3; 3.77	3.2; 3.68	4.5; 4.49	4.7; 4.37	4.25; 4.63
Amount of postoperative change of acetabular correction in the first year (AI)	2.4; 5.0	2.3; 4.96	3.3; 5.27	2.2; 3.46	4.9: 6.74

Interobserver reliability between observers 1 and 2, assessed with ICC, resulted in excellent values for almost all measurements [14]. The distribution of the values was very similar to those of observer 1, underlining the conclusion

## Discussion

The two most important findings of this examination were: First, increasing age and increasing magnitude of acetabular correction negatively influence bone healing in TPO. Second, the measurable amount of a slight change in the postoperative correction, synonymous with a micromotion, does not have a significant impact on bone healing in TPO.

In the current literature, there is rather little scientific evidence about the risk factors affecting the bone healing process after TPO. Yilmaz et al. conducted a retrospective case–control study, comparing 53 patients suffering from a non-union with 117 patients who did not experience non-union [15]. These cases were identified out of a total of 3269 patients who had undergone TPO. The pelvic osteotomy was performed according to the technique described by Tönnis and Kalchschmidt, in a manner comparable to the procedure in the present examination. The authors investigated a similar risk profile as in the present study. A significant difference between age and the degree of surgical correction, expressed by the postoperative LCEA, was detected in the non-union and the union group. Interestingly, in the present study, a very similar risk profile showed a significant association with a non-union: patients’ age to the time of TPO and the magnitude of surgical correction, expressed by the difference of pre- and postoperative LCEA. The latter can be explained by decreased contact surfaces at the osteotomy sites due to the large rotational movement required to perform a large correction [16]. Contrary to our expectations, an increase in BMI and the abuse of nicotine did not show a significant association with a non-union. Pearson’s chi-square test showed a significant relationship between the risk factor “wound healing disorder” and non-union. Overall, seven cases with a wound healing disorder were documented, four in the cohort of the non-unions. An association between a wound-healing disorder and the occurrence of a non-union seems probable. However, with respect to the numbers, the statistical power is low.

In the present examination, the progress of bone healing was determined 1 year after TPO by the state of a union or a non-union. Although reporting on patients undergoing Bernese Periacetabular Osteotomy (PAO), in a recent examination, Selberg et al. investigated what proportion of patients experienced complete bony healing versus non-union during the first year [17]. A total of 286 patients had undergone PAO to treat symptomatic acetabular dysplasia and were eligible for the study. In this group of patients, at a minimum of twelve months after PAO, the proportion of non-union was 8%. Six months after PAO, fewer than half of the patients had complete healing of the osteotomies. However, the authors stated that more than 90% of the patients can expect to have completely healed osteotomy sites at 1 year or more postoperatively. Therefore, Selberg et al. advised surgeons to avoid unnecessary interventions if a non-union is observed at 6 months postoperatively.

Even though Selberg et al. reported on the results after PAO, in our examination, a similar behaviour of osteotomy healing was observed after TPO: At the radiographic follow-up after 1 year more than 90% of the cases showed a complete consolidation. Non-union was detected in 8.5% of the cases, amongst these 5% which did not report relevant symptoms. The latter cases predominantly showed a non-union solely at one osteotomy site. In these cases, likewise, to the completely consolidated pelvic osteotomies, implant removal was performed around one year after TPO without any further interventions. In eight cases (3.6%), the patients suffered from non-unions. The consecutively performed computer-tomography (CT) scan of the pelvis mostly revealed a non-union in one or even two of the three osteotomies. In the majority of these cases, revision of the osteotomy sites was performed, including transplantation of autologous cancellous bone from the iliac crest and additional osteosynthesis with a fully threaded 4.5 mm steel screw.

In addition, Selberg et al. described that more-severe acetabular dysplasia and older age were associated with non-union, with the latter being a predictor of non-union at 12 months postoperatively. Well knowing the technical differences between PAO and TPO, the results of Selberg underline the findings in the present examination, regarding the risk profile for a non-union after pelvic osteotomy and the dynamics of bone healing in the first year.

In the present work, an extensive radiographic assessment of five consecutive pelvic radiographs (day of surgery, 5 days, 6 weeks, 3 months and 1 year after surgery) made a very slight change in acetabular correction recognizable. The radiographic analysis was performed by two experienced observers (DD and BL) for every case. The analysis showed that throughout one year after surgery, the mean LCEA increased by 1.6° (DD) and AI decreased by 1.3° (3.3° and 2.4° for BL, respectively). This suggests the presence of a settling behavior of the acetabular fragment, involving a slight emphasis of the initial correction. In the subgroups of the non-union cases, the micromotion in the acetabular fragment was observed to even be slightly higher (Tables 3, 4). This, in turn, suggests a relationship between a slightly enhanced micromotion and a disturbance in bone healing. Interestingly, almost all of the fragment settling took place in the first 3 months after surgery, in a sensible phase of bone healing. This might have been of interest to predict further consolidation of the osteotomy sites. However, statistical analysis could not confirm that the amount of change of LCAE or AI correlates with the occurrence of a non-union. Most likely, a certain micromotion of the acetabular fragment induces compression forces onto the osteotomy sites, which promotes bone healing. For a reliable statement, when there is too much acetabular micromotion for undisturbed bone healing, future research is necessary.

This examination has several limitations: first, due to the analysis of plane pelvic radiographs, the slight acetabular settling behavior was assessed solely in one plane, disregarding, for example, a potential antero-posterior micromotion. On the other hand, against the background of radiation protection, it would be very difficult to obtain repeated CT scans of the pelvis from a large number of individuals. Second, the assessment of antero-posterior acetabular orientation was not taken into consideration. With the knowledge, that parameters expressing the antero-posterior acetabular version, e. g. the anterior and posterior wall indices or the crossing-sign are susceptible to a tilt or malrotation of the pelvic radiograph, only LCEA and AI were measured. These parameters have been well described and have proven to be robust against small changes in the patient positioning for the radiographic examination.

## Conclusion

The age at the time of TPO and the magnitude of acetabular correction negatively influenced the healing progress of the osteotomy sites. This should be taken into account in the decision-making for TPO and in the information on the operation, when the patient is slightly older or when a large acetabular correction is foreseeable. The rates and the risk factors of observed non-unions were comparable to those described for periacetabular osteotomy. The assessment of a series of identically performed pelvic radiographs in the follow-up after TPO over the course of 1 year, allowed us to detect a postoperative settlement behavior of the acetabular fragment. The amount of slight change of LCEA and AI, predominately measurable in the first 12 weeks after TPO, did not correlate with a disturbance of bone healing. Future examination will be necessary to assess to what extent a micromotion of the acetabular fragment is beneficial for osteotomy healing and when it is not anymore.

## Data Availability

The authors agree to deposit the data that support the findings of this examination. The data have not been uploaded to a public repository yet.
